# Dihydrocapsaicin Inhibits Epithelial Cell Transformation through Targeting Amino Acid Signaling and c-Fos Expression

**DOI:** 10.3390/nu11061269

**Published:** 2019-06-04

**Authors:** Ji Su Lee, Yeong A. Kim, Young Jin Jang, Yongtaek Oh, Sanguine Byun

**Affiliations:** 1 Division of Bioengineering, Incheon National University, Incheon 22012, Korea; 201821148@inu.ac.kr; 2 Nongshim Co., Ltd, 112, Yeouidaebang-ro, Dongjak-gu, Seoul 07057, Korea; 0nakya0@naver.com; 3 Department of Agricultural Biotechnology, College of Agriculture and Life Sciences, Seoul National University, Seoul 08826, Korea; 4 Korea Food Research Institute, Wanju-gun, Jeollabuk-do 55365, Korea; jyj616@kfri.re.kr; 5 Department of Diagnostics, College of Korean Medicine, Woo-suk University, Wanju-gun, Jeollabuk-do 55338, Korea; ohyt@woosuk.ac.kr

**Keywords:** dihydrocapsaicin, cell transformation, chili pepper, amino acid, c-Fos, mTOR

## Abstract

Chili peppers are one of the most widely consumed spices worldwide. However, research on the health benefits of chili peppers and some of its constituents has raised controversy as to whether chili pepper compounds possess cancer-promoting or cancer-preventive effects. While ample studies have been carried out to examine the effect of capsaicin in carcinogenesis, the chemopreventive effect of other major components in chili pepper, including dihydrocapsaicin, capsiate, and capsanthin, is relatively unclear. Herein, we investigated the inhibitory effect of chili pepper components on malignant cell transformation. Among the tested chili pepper compounds, dihydrocapsaicin displayed the strongest inhibitory activity against epidermal growth factor (EGF)-induced neoplastic transformation. Dihydrocapsaicin specifically suppressed EGF-induced phosphorylations of the p70S6K1-S6 pathway and the expression of c-Fos. A reduction in c-Fos levels by dihydrocapsaicin led to a concomitant downregulation of AP-1 activation. Further analysis of the molecular mechanism responsible for the dihydrocapsaicin-mediated decrease in phospho-p70S6K1, revealed that dihydrocapsaicin can block amino acid-dependent mechanistic targets of rapamycin complex 1 (mTORC1)-p70S6K1-S6 signal activation. Additionally, dihydrocapsaicin was able to selectively augment amino acid deprivation-induced cell death in mTORC1-hyperactive cells. Collectively, dihydrocapsaicin exerted chemopreventive effects through inhibiting amino acid signaling and c-Fos pathways and, thus, might be a promising cancer preventive natural agent.

## 1. Introduction

Chili pepper (fruits from the plants of *Capsicum*) is a widely consumed spice in various countries with multiple studies reporting its impact on health. Chili pepper components can be divided into capsaicinoids, capsinoids, and carotenoids. The most abundant capsaicinoids in chili peppers are capsaicin (N-[(4-hydroxy-3-methoxypheny) methyl]-8-methyl-E-6-nonenamide) and dihydrocapsaicin (N-[(4-hydroxy-3-methoxyphenyl)methyl]-8-methyl-6-nonanamide) [1]. Capsinoids include capsiate, dihydrocapsiate, and nordihydrocapsiate [2]. Carotenoids are major sources responsible for the red color in chili peppers, with capsanthin contributing the highest portion in most of the varieties [3]. Chili pepper and its constituents are reported to exert pain relief, anti-inflammatory, anti-oxidative, anti-obesity, and anti-cancer effects [2,4,5]. Studies on the cancer preventive/therapeutic effects of chili pepper mostly focus on capsaicin and other chili pepper compounds have a relatively limited literature regarding their bioactivity [2]. More importantly, multiple lines of evidence suggest conflicting data on the role of capsaicin during carcinogenesis [6,7,8]. Chili peppers also contain high amounts of compounds such as dihydrocapsaicin, capsiate, or capsanthin, which might contribute to the health-promoting effects of chili peppers. Therefore, studying the effects of other chili pepper components in cancer development will aid in fully understanding the influence of chili pepper consumption.

The mammalian target of rapamycin (mTOR, or mechanistic target of rapamycin) is a serine/threonine kinase that belongs to the phosphatidylinositol 3-kinase-related protein kinase family [9]. The mTOR signaling network senses various environmental cues, including growth factors, amino acids, and stress levels, and executes subsequent cellular activities through two distinct multiprotein complexes: mTOR complex 1 (mTORC1) and mTOR complex 2 (mTORC2) [9]. mTORC1 regulates translation, cell proliferation, and growth by activating downstream effectors such as p70S6K1 and 4E-BPs. mTORC2 controls cell survival and metabolism mainly through activating Akt [10]. As the mTOR pathway plays a critical role in cell survival and proliferation, various cancers have been reported to have elevated mTOR activity [11]. Therefore, targeting the mTOR pathway is a promising strategy for cancer prevention and therapy [9,10,11].

c-Fos is a proto-oncogene which promotes malignant conversion, tumor formation, invasion, and metastasis [12,13]. Many studies have reported the overexpression of c-Fos in human cancers and its correlation with poor prognosis in patients [14,15,16]. Expression of c-Fos induces tumorigenesis, while deficiency of c-Fos can prevent cancer development and cancer progression [17,18]. Also, overexpression of c-Fos has been implicated in resistance to cancer therapy and enhancement of cancer stem cell stemness [14,19]. Hence, inhibiting c-Fos can be an attractive approach for cancer prevention.

In the present study, to draw direct comparisons among major chili pepper constituents, colony formation was analyzed after cells were treated with epidermal growth factor and chili pepper compounds. Dihydrocapsaicin displayed the strongest inhibitory effect against malignant cell transformation. As the cancer preventive effect and molecular mechanism of dihydrocapsaicin is not well understood, we explored the mechanism of action to understand the potential of dihydrocapsaicin as an anti-cancer agent.

## 2. Materials and Methods

### 2.1. Materials

Dihydrocapsaicin was obtained from Cayman Chemical (Ann Arbor, MI, USA). Capsiate, capsanthin, capsaicin, 12-O-tetradecanoylphorbol 13-acetate (TPA), glutaraldehyde, crystal violet, glutamine, gentamicin, and β-actin antibody were purchased from Sigma-Aldrich (St. Louis, MO, USA). Eagle’s MEM was purchased from Corning (New York, NY, USA). Antibodies for phospho-Akt, phospho-p38, phospho-JNK, phospho-p90RSK, phospho- phospho-p70S6K, phospho-S6, Akt, p70S6K, c-Fos, and p38 were obtained from Cell Signaling Technology (Danvers, MA, USA). Antibodies to detect phosphorylated ERK1/2, ERK1/2, and RSK2 were purchased from Santa Cruz Biotechnology (Dallas, TX, USA).

### 2.2. Cell Culture

The JB6 P+ cell line was cultured in 5% FBS MEM at 37 °C supplemented with 1% penicillin/streptomycin (Corning, New York, NY, USA) in a humidified chamber with 5% CO_2_. The *TSC^+/+^ p53^−/−^*and *TSC^−/−^ p53^−/−^* mouse embryonic fibroblast cell line was cultured at 37 °C in Dulbecco Modified Eagle Medium (DMEM, Corning, New York, NY, USA) supplemented with 10% FBS (Gibco, Waltham, MA, USA) and 1% penicillin/streptomycin (Corning, New York, NY, USA).

### 2.3. Cell Viability

Cell viability was analyzed by counting the cell numbers using trypan blue. Cells were starved overnight (0.1% FBS) and then the samples were treated for 24 h in 0.1% FBS MEM. Viable cells were measured by Countess II FL Automated Cell Counter (Thermo Fisher Scientific, Waltham, MA, USA).

### 2.4. Cell Transformation Assay

The effect of samples against epidermal growth factor (EGF)- or TPA-induced cell transformation was examined as described before [20]. An agar mixture was made with basal medium eagle (Sigma-Aldrich, St. Louis, MO, USA), 10% FBS, glutamine, gentamicin, PBS, and agar. JB6 P+ cells are sensitive to tumor promoter-mediate transformation, and thus are widely used to study the process of neoplastic cell transformation and carcinogenesis [20,21]. JB6 P+ cells (8000 cells/well) were treated with or without the samples and EGF/TPA in the agar mixture. The agar mixture was dropped to each well in a 6-well plate and left in RT for 1hr to solidify. Then the agar plates were maintained in an incubator with 5% CO_2_ at 37 °C for 14–20 days. The images of the colonies were counted using the Image-Pro Plus software (Media Cybernetics, Rockville, MD, USA).

### 2.5. Luciferase Assays

AP-1 and COX-2 luciferase reporters were stably transfected to the JB6 P+ cell line and maintained in media supplemented with G418 [22]. Dihydrocapsiain was treated to cells 1 h before the EGF (10 ng/mL) treatment. Cells were disrupted and the luciferase activity was measured with Varioskan Lux Multimode Microplate Reader (Thermo Fisher Scientific, Waltham, MA, USA).

### 2.6. Immunoblot Assay 

Cells were rinsed, scraped off and lysed using RIPA buffer with a protease and phosphatase inhibitor cocktail (Sigma–Aldrich, St. Louis, MO, USA). After centrifugation of the lysate, the supernatants were collected and quantified using a dye-binding protein assay kit (Bio-Rad, Hercules, CA, USA) or the Pierce BCA Protein Assay Kit (Thermo Fisher Scientific, Waltham, MA, USA). Proteins were separated by 10% SDS-PAGE and transferred to a nitrocellulose membrane (Bio-Rad, Hercules, CA, USA). After blocking in 5% skim milk in TBS, containing 0.1% Tween 20 (TBST), corresponding antibodies were applied to membranes and incubated overnight at 4 °C. After washing with TBST, a HRP-conjugated secondary antibody was applied to the membranes and bands were visualized using Western lightning Plus-ECL (PerkinElmer, Waltham, MA, USA).

### 2.7. AP-1 Transcription Activity Assay

The activity of c-Fos and p-c-Jun was assessed using the TransAM™ AP-1 family transcription assay kit (Active Motif, Carlsbad, CA, USA) as previously described [20]. The DNA binding activity of AP-1 factors were measured according to the manufacturer’s instructions by ELISA. Cell extracts were added to a 96-well plate coated with TPA response element (TRE; 5′-TGAGTCA-3′), which can bind c-Jun and c-Fos. After washing, the plate was incubated with antibodies for 1 h. Next, the secondary HRP-conjugated antibody was applied and the absorbance was measured.

### 2.8. Immunofluorescence Assay

Immunofluorescence to detect mTOR translocation was performed as previously described [23]. *TSC*2^−/−^ *p53^−/−^* MEFs (kindly provided by Dr. John Blenis, Weill Cornell Medical College) were seeded onto fibronectin-coated chamber slides. Cells were starved for serum over night and deprived of amino acids for 4 h using a media without any amino acids. After re-stimulating with amino acid for 1 h, the cells were fixed with 4% formaldehyde, and, subsequently, permeabilized using 0.05% saponin in PBS. Slides were treated with blocking solution (5% bovine serum albumin), and incubated with primary antibodies (anti-mTOR: Cell signaling, anti-LAMP1: BD pharmingen, San Jose, CA, USA) overnight at 4 °C followed by secondary antibodies conjugated with Alexa488 and Alexa568. Images were captured with Carl Zeiss LSM700 confocal laser scanning microscope and measured using ZEN microscope software.

### 2.9. Analysis of Cell Death 

After cell seeding (2.2 × 10^5^/mL), FBS (Gibco, Waltham, MA, USA), 10% Dialyzed FBS (Gibco, Waltham, MA, USA), DMEM (Welgene, Gyeongsan-si, Korea) and a medium without amino acid DMEM (Welgene, Gyeongsan-si, Korea) were used in order to make media with 100%, 50%, and 0% amino acid. The medium was changed according to each condition. Dihydrocapsaicin was treated for 48 h. Cell death was determined using the Countess II FL automated cell counter, (Thermo Fisher Scientific, Waltham, MA, USA) after trypan blue staining. Cell counts were performed in triplicate.

### 2.10. Colony Formation Assay

Colony formation was measured based on a previously reported protocol [24]. Cells were seeded in a 6-well plate and treated with dihydrocapsaicin with or without EGF in media with 2.5% FBS. The colonies were fixed and stained using 6.0% glutaraldehyde and 0.5% crystal violet solution. Colonies were quantified by dissolving the dye in 10% acetic acid and measuring the absorbance at 590 nm.

### 2.11. Statistical Analysis 

Bar graphs are expressed as means ± S.D., and analysis of variance was used for statistical comparisons. Statistical significance was determined using *p* < 0.05 as a threshold. Statistical Analysis Software (SAS Inc, Cary, NC, USA) was used.

## 3. Results

### 3.1. Dihydrocapsaicin Suppresses EGF- and TPA-Mediated Neoplastic Cell Transformation

**Figure 1:**
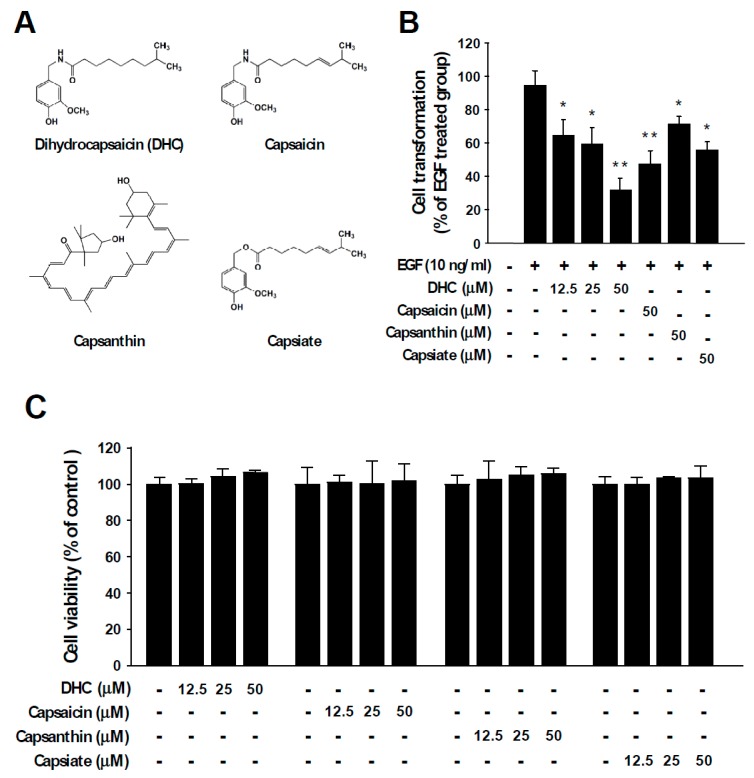
Chili pepper compounds inhibit epidermal growth factor (EGF)-mediated neoplastic transformation. (**A**) Chemical structures of dihydrocapsaicin (DHC), capsaicin, capsanthin, and capsiate. (**B**) EGF-induced colony formation in soft agar was used to assess anchorage-independent growth. JB6 P+ cells were incubated in soft agar with the indicated compounds and EGF. Colonies were automatically counted 14 days later under a microscope using the Image-Pro Plus program. The number of colonies was counted and expressed in relative terms compared to the EGF-only treated group. Data are presented as means ± S.D. of triplicate samples from three independent experiments. The asterisks (* *p* < 0.05 and ** *p* < 0.01) indicate significant differences between a group treated with EGF alone and co-treated with EGF and a chili pepper compound. (**C**) The effect of chili pepper compounds on the cell viability of JB6 P+ cells. Cells were serum starved and treated with the compounds at the indicated concentrations. Cell viability was measured as described in the Materials and Methods section.

Epidermal growth factor (EGF) stimulates cell growth and abnormal EGF signaling is known to promote malignant transformation, cancer progression, and metastasis [25,26]. EGF functions as a ligand to activate the EGF receptor (EGFR) and dysregulation of EGF and/or EGFR is known to cause various types of human cancers [27,28,29]. To examine the chemopreventive potential of chili pepper components (Figure 1A), we investigated their inhibitory activities against neoplastic cell transformation induced by EGF stimulation. Anchorage-independent growth in agar was examined after chili pepper compounds and EGF were treated to JB6 P+ cells. The number of colonies formed in the agar was measured. Among the tested compounds, dihydrocapsaicin (DHC) exhibited the strongest protective effect against EGF-induced neoplastic cell transformation (Figure 1B). At identical concentrations, capsaicin, capsanthin, and capsiate reduced cell transformation by 50%, 25%, and 41%, respectively (Figure 1B). DHC also suppressed 12-O-tetradecanoylphorbol 13-acetate (TPA)-induced cell transformation (Figure S1). In addition, DHC treatment led to a reduction in 2D colony formation in a dose-dependent manner (Figure S2). The chili pepper components DHC, capsaicin, capsanthin, and capsiate did not show significant cytotoxicity (Figure 1C). These results suggest that dihydrocapsaicin can act as a potent inhibitor of neoplastic cell transformation.

### 3.2. Dihydrocapsaicin Suppresses p70S6K1 Phosphorylation and c-Fos Expression

**Figure 2:**
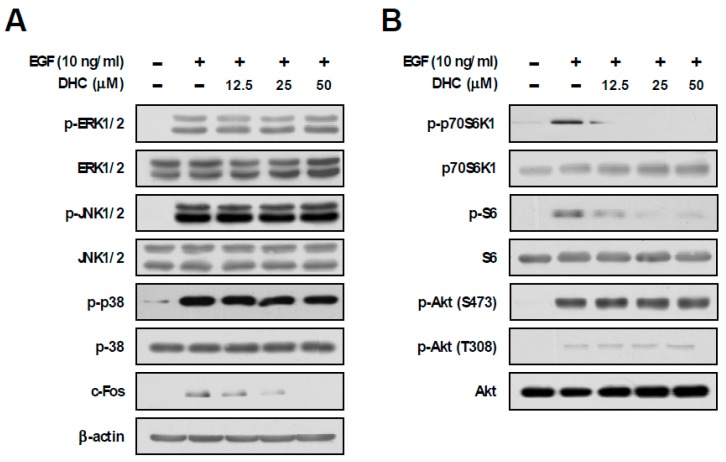
Effect of dihydrocapsaicin on EGF-induced MAPKs and mTOR pathway. (**A**,**B**) JB6 P+ cells were starved using 0.1% FBS-MEM for 24 h and then treated with dihydrocapsaicin 1 h prior to EGF (10 ng/mL) treatment. Cells were collected 15 min after EGF (10 ng/mL) treatment, and immunoblot analysis was performed using the corresponding antibody.

In order to understand the underlying molecular mechanism for the chemopreventive effect of DHC, we examined the downstream signaling pathways mediated by EGF. The MAPKs and mTOR signaling pathways have been reported to play crucial roles in EGF-mediated cancer development [30]. Treatment with DHC did not cause any noticeable effects against EGF-induced phosphorylations of ERK, JNK, and p38, whereas c-Fos expression was downregulated in a dose-dependent manner (Figure 2A). Additionally, DHC decreased EGF-induced phosphorylations of p70S6K1 and S6, while not affecting the phosphorylation of Akt (Figure 2B). When activated, mTORC1 phosphorylates p70S6K1, whereas mTORC2 phosphorylates Akt (S473) [11]. Therefore, DHC appears to selectively suppress the mTORC1 signaling pathway.

### 3.3. Dihydrocapsaicin Attenuates EGF-Induced c-Fos and AP-1 Activities and COX-2 Transcriptional Activity

**Figure 3:**
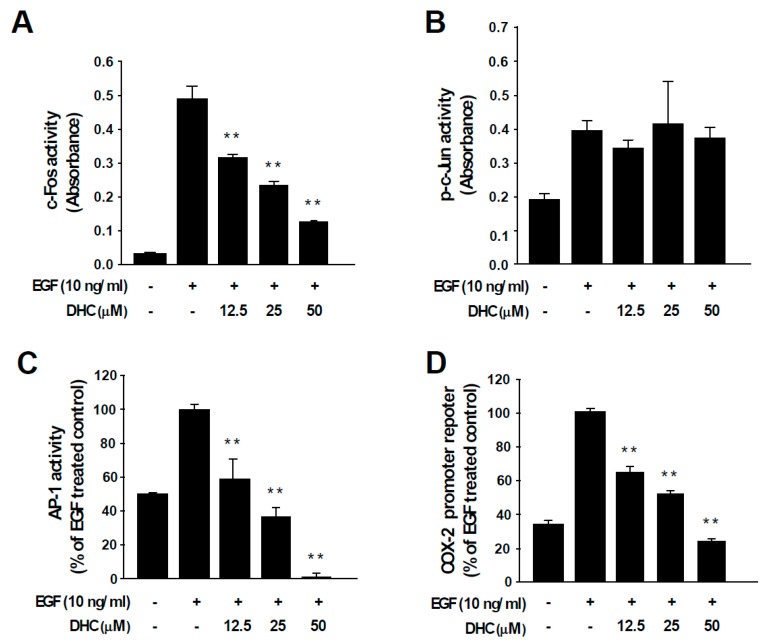
Effect of dihydrocapsaicin on EGF-induced c-Fos, c-Jun, and AP-1 activities and COX-2 promoter activity. (**A, B**) TransAM™ AP-1 family transcription assay kit was used to assess c-Fos and c-Jun activity. Nuclear protein extracts from JB6 P+ cells were used after the indicated treatment. Data are presented as means ± S.D. (**C**) JB6 P+ cells stably expressing an AP-1 luciferase reporter plasmid were treated as indicated. (**D**) JB6 P+ cells stably expressing COX-2 promoter reporter plasmid were treated as indicated. ** *p* < 0.01, significant differences between the group treated with EGF alone and co-treated with dihydrocapsaicin and EGF.

AP-1 transcription factors are dimeric proteins composed of the Fos and Jun families [12]. The activity of AP-1 controls key aspects of carcinogenesis, including cell proliferation, differentiation, survival, and neoplastic transformation [12]. c-Fos functions as a potent oncogenic protein by participating as a major member of AP-1 during cancer development [12,13]. Treatment with DHC suppressed c-Fos activity (Figure 3A), which was in line with the reduced protein expression (Figure 2A). In contrast, DHC treatment did not affect the activity of c-Jun, suggesting that DHC specifically targets c-Fos (Figure 3B). More importantly, DHC treatment completely blocked EGF-induced AP-1 activity (Figure 3C). Next, we assessed the effect of DHC on COX-2 promoter activity. COX-2 is an important inflammatory mediator of cell transformation that is transcriptionally controlled by AP-1 [31]. EGF-induced COX-2 promoter activity was attenuated by DHC (Figure 3D). These results indicate that DHC blocks AP-1 via c-Fos downregulation which may, subsequently, lead to the inhibition of COX-2 and neoplastic transformation.

### 3.4. Dihydrocapsaicin Targets the Amino Acid Signaling Pathway

**Figure 4:**
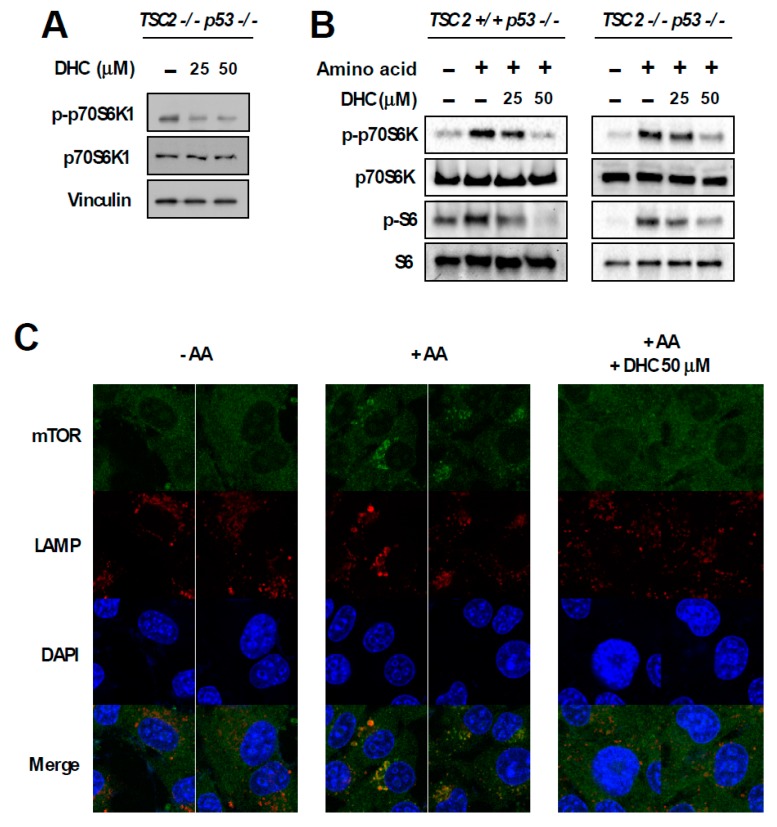

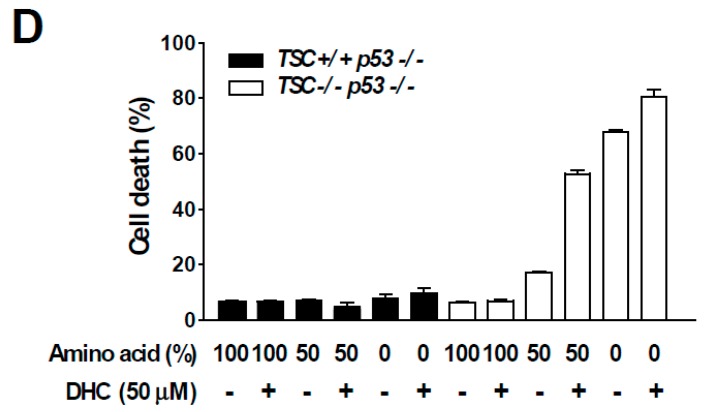
Effect of dihydrocapsaicin on amino acid signaling. (**A**) TSC2^−/−^, p53^−/−^ MEFs were treated as indicated for 30 min and lysed for immunoblot analysis. Media was changed to a serum-free media when dihydrocapsiain was added. (**B**) TSC2^+/+^, p53^−/−^ MEFs were incubated with amino acid-deprived and dialyzed-FBS containing media for 5 h to provide amino acid starvation. Dihydrocapsaicin was pre-treated for 1 h and cells were subsequently re-stimulated with amino acid, using media containing amino acid and regular FBS for 1 h. TSC2^−/−^, p53^−/−^ MEFs were serum starved overnight and then incubated with amino acid-deprived and serum-free media for 5 h. Dihydrocapsaicin was pre-treated for 1 h and cells were subsequently re-stimulated with amino acid, using serum-free media containing amino acid for 1 h. (**C**) Immunofluorescence was performed to examine the translocation of mTOR in TSC2^−/−^, p53^−/−^ MEFs. Mechanistic targets of rapamycin (mTOR) is shown in green and the lysosomal marker, lysosomal-associated membrane protein 1 (LAMP1) is shown in red. DAPI (4′,6-diamidino-2-phenylindole) is shown in blue. (**D**) 24 h after seeding, media was changed to regular growth media, or media with reduced amino acid content as indicated. Cell death was measured 48 h after changing the media.

In order to dissect the molecular mechanism responsible for DHC-mediated inhibition of phospho-p70S6K1, we utilized TSC2 knock-out (KO) MEFs. The TSC1-TSC2 complex is a key negative regulator of mTORC1. As the TSC1-TSC2 complex transfers majority of the upstream signaling pathways controlling mTORC1 activity [10], we questioned whether DHC modulates mTORC1 activity via the TSC complex. Interestingly, DHC suppressed the phosphorylation of p70S6K1 in TSC2 KO MEFs, suggesting that the DHC inhibits mTORC1 signaling in a TSC-independent manner (Figure 4A). In addition, we conducted in vitro kinase assays on p70S6K1, PKBα, PKBβ, and SGK using DHC and found that DHC does not target these kinases, implying that reduction in phosphorylation of p70S6K1 by DHC is not a result of directly suppressing the activity of these kinases (Figure S3). TSC KO MEFs display a hyperactive-mTORC1 phenotype in a TSC complex-independent manner, which provides a useful environment to study the relationship between amino acid and mTORC1 activity [32]. The activation of mTORC1 requires several necessary conditions, and input from sufficient amino acid levels is known to play a critical role in mTORC1 signal transduction [11]. Hence, we next examined whether DHC affects amino acid-mediated mTORC1 activation. Cells were starved from all amino acids and re-stimulated to investigate the effect of DHC on amino acid signaling. Pre-treatment with DHC was able to inhibit the amino acid-induced phosphorylation of p70S6K1 and S6 in both TSC WT and KO MEFs (Figure 4B), suggesting that DHC targets the amino acid-mediated mTORC1 activation pathway. To further confirm this phenomenon, we analyzed mTOR translocation. The presence of amino acid is not only a necessary condition for mTORC1 activation, but also an inducer of mTORC1 translocation to the lysosome [33]. While amino acid-starved cells exhibited a dispersed pattern of mTOR, the addition of amino acid induced the mTOR translocation to the lysosome (Figure 4C). Pre-treatment with DHC prevented the amino acid-mediated mTOR translocation, demonstrating that DHC can block amino acid signals leading to mTORC1 activation (Figure 4C). Previous reports demonstrated that hyperactive mTORC1 cells are sensitive to amino acid starvation [34]. Thus, we questioned whether DHC can promote cell death in cells with high mTORC1 activity. In consistence with previous studies, TSC2 WT MEFs (low-mTORC1) were relatively resistant to amino acid deprivation, whereas TSC2 KO MEFs (high-mTORC1) were sensitive to the removal of amino acids (Figure 4D). Strikingly, the addition of DHC was able to selectively promote cell death in TSC2 KO MEFs (high-mTORC1) with reduced amino acid levels, while showing little cytotoxicity towards TSC2 WT MEFs (low-mTORC1) (Figure 4D). These results show that in addition to repressing amino acid signaling, DHC can also augment cell death mediated by amino acid deprivation in mTORC1-hyerpactive cells.

## 4. Discussion

Although there have been previous studies reporting the cytotoxic effects of DHC against cancer cells [35,36,37], the preventive potential of DHC against carcinogenesis under non-cytotoxic concentrations, has been largely unknown. In the present study, we evaluated the chemopreventive potential of chili pepper components and discovered that DHC can suppress EGF-induced neoplastic transformation more effectively than other chili pepper compounds, such as capsaicin, capsanthin, and capsiate. Investigation of the molecular mechanism revealed that DHC can downregulate c-Fos expression and inhibit mTORC1 activity by targeting the amino acid signaling pathway. In addition, DHC augmented amino acid reduction-mediated cell death, specifically in mTORC1-hyperactive cells. Capsaicin and DHC share similar structures but DHC displays superior inhibitory effects against neoplastic transformation. There have been many reports that demonstrated the differential impact on bioactivity depending on small differences in the chemical structure [38,39,40]. The flexibility provided by removal of the double bond would likely be a major reason that enables DHC to interact with certain targets better than capsaicin. Further studies on the structure–activity relationship would help understand a more detailed mechanism of action.

c-Fos and mTOR are well-known key players in carcinogenesis, and the effect of its inhibition has been shown by many previous reports to critically control cancer development. While the overexpression of c-Fos is sufficient to cause cell transformation [17,41], deficiency in c-Fos can block tumorigenesis [18]. Furthermore, the suppression of mTORC1 activity has been shown to be effective in preventing tumorigenesis in a variety of models [42,43,44,45]. We observed that DHC can block both c-Fos and mTORC1 pathways. Suppressing c-Fos and mTORC1 signaling has been reported to show preventive effects in various types of cancer models. DHC could be expected to display broad chemopreventive activities against diverse carcinogenic conditions.

We found that DHC can specifically inhibit the amino acid-induced mTORC1 activity. The mTORC1 pathway plays a key role in cancer development and progression [10,46]. Most of the known inhibitors of the mTORC1 pathway target the growth factor-driven axis which controls mTOR activity via the TSC complex. However, mTORC1 also functions as an amino acid sensor, and the level of amino acid is a critical necessary condition of mTORC1 activation [10,46]. Only a few compounds are reported to affect the amino acid signaling pathway [47,48]. DHC is the first phytochemical and food compound to be reported to target amino acid signaling. Although DHC treatment alone did not cause cytotoxicity towards mTORC1-hyperactive cells, when amino acids were reduced or completely deprived, adding DHC increased the cytotoxic effects. As DHC was able to raise cell death levels even when amino acids were fully removed from the media, it is likely that the additional cell death could at least partially be attributed to DHC’s ability to suppress c-Fos expression. These results suggest that DHC could be useful in targeting malignancies with high mTORC1 activity. Further studies on dissecting the detailed mechanism of how DHC suppresses amino acid signaling could aid in improving our knowledge of the relationship between amino acid and the mTOR pathway.

There have been conflicting reports on whether chili pepper promotes or prevents carcinogenesis. Many studies suggest the chemopreventive/chemotherapeutic potential of chili pepper based on the ability of capsaicin to induce apoptosis or inhibit proliferation in cancer cells [49]. However, multiple studies report capsaicin to be a causation of cancer. Capsaicin has been reported to be mutagenic or induce tumor formation in several animal studies [7,8,50]. Results from human studies also show a controversial role of chili pepper in carcinogenesis [6]. In addition to the complicated difference in genetic backgrounds, the type of chili pepper consumed could be an important variable in determining the effect of chili peppers in cancer development. Depending on the chili pepper type, the amount and ratio of DHC, capsaicin, capsiate, and other components, can vary significantly [51]. Our research shows that at identical conditions, DHC has stronger chemopreventive activity compared to other major chili pepper compounds. Considering that DHC is one of the most common components in chili peppers, the content of DHC, in addition to capsaicin, should also be considered when evaluating the impact of chili pepper consumption in cancer. In addition, DHC is not only found in chili peppers but can also be found in paprika [52]. Further identification of the food sources that contain DHC and developing processing methods in order to optimize DHC content could aid in providing higher levels of DHC that may reduce the risk of cancer development.

## Supplementary Materials

The following are available online at https://www.mdpi.com/2072-6643/11/6/1269/s1, Figure S1: DHC inhibits TPA-induced cell transformation, Figure S2: DHC inhibits colony formation, Figure S3: Effect of DHC on p70S6K1, PKB, and SGK kinase activity in vitro.
