# Supplementary material for: Dihydrocapsaicin Inhibits Epithelial Cell Transformation through Targeting Amino Acid Signaling and c-Fos Expression
Source: Nutrients. 2019 Jun 4;11(6):1269. doi: 10.3390/nu11061269 (PMC6627986; doi:10.3390/nu11061269)
Supplement: Supplementary file 1 [file nutrients-11-01269-s001.pdf]

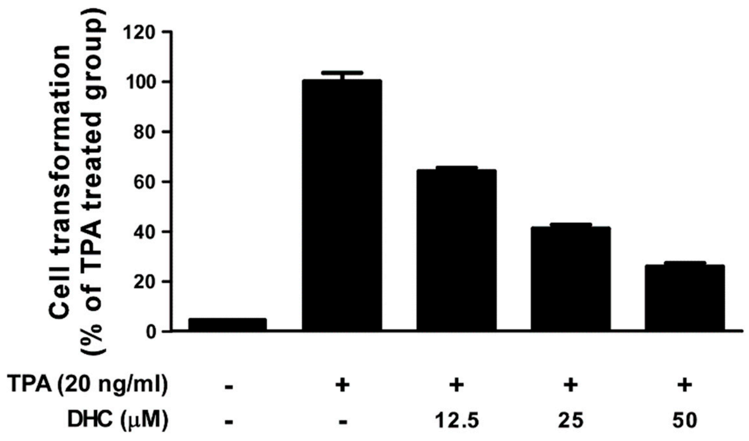

Figure S1. DHC inhibits TPA-induced cell transformation.

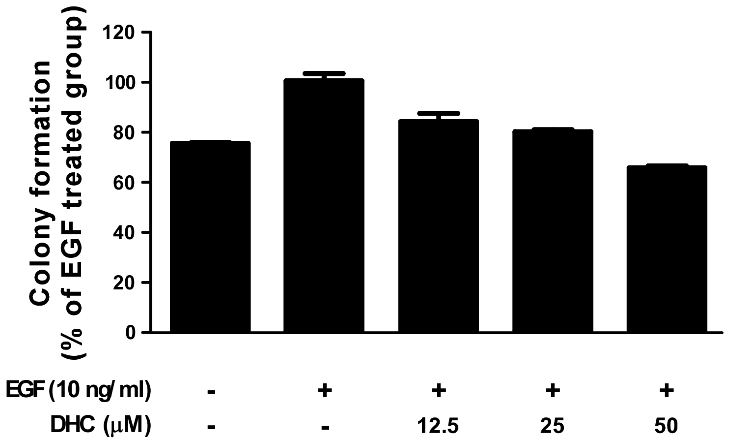

Figure S2. DHC inhibits colony formation.

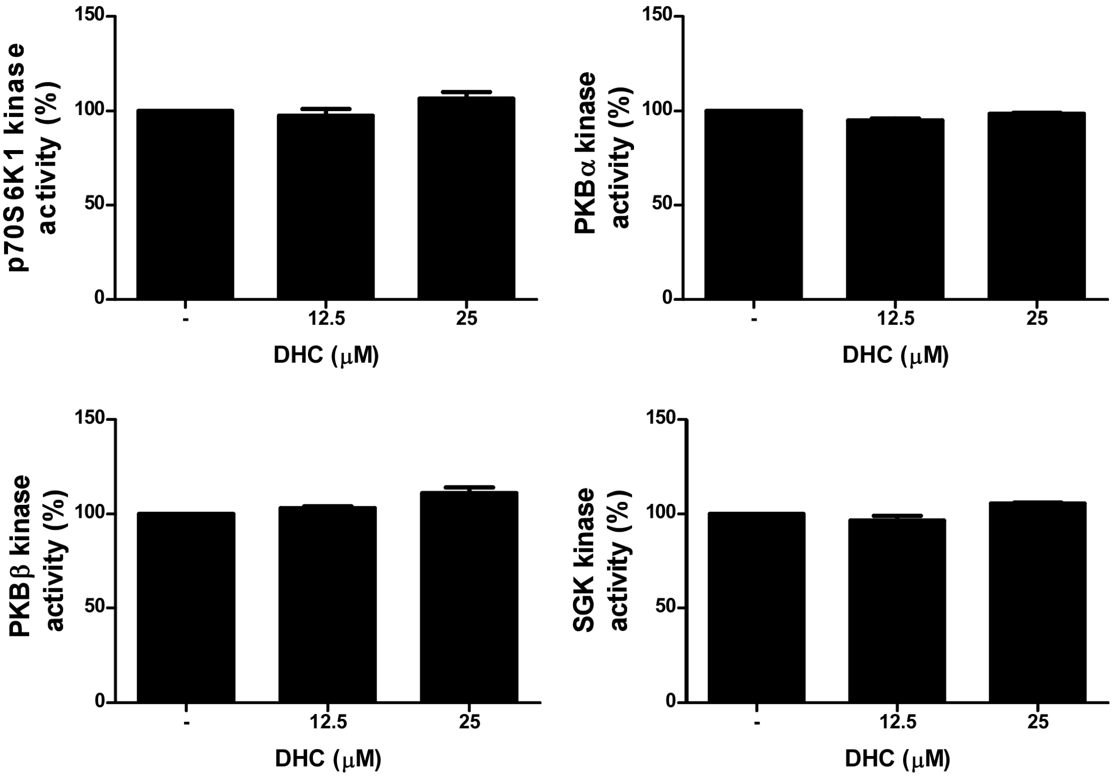

Figure S3. Effect of DHC on p70S6K1, PKB, and SGK kinase activity in vitro.
